# Urbanisation Leaves a Genetic Signature but No Detectable Demographic Effects in a Small Mammal Population

**DOI:** 10.1002/ece3.74273

**Published:** 2026-10-05

**Authors:** Sinah Drenske, Conny Landgraf, Alina Theresa Berger, Alina Doreen Stemmer, Aimara Planillo, Johanna Leona Eul, Bianca Wist, Kathleen Röllig, Ashlee Jean Mikkelsen, Melanie Dammhahn, Jörns Fickel, Stephanie Kramer‐Schadt

**Affiliations:** ^1^ Leibniz Institute for Zoo and Wildlife Research (IZW) Berlin Germany; ^2^ Institute of Ecology, Chair of Applied Animal Ecology Technische Universität Berlin Berlin Germany; ^3^ Department of Biology, Chemistry, Pharmacy Freie Universität Berlin Berlin Germany; ^4^ Estación Biológica de Doñana (EBD) Spanish National Research Council (CSIC) Seville Spain; ^5^ Institute for Biochemistry and Biology, Animal Ecology University of Potsdam Potsdam Germany; ^6^ Functional Ecology, Institute of Cell and Systems Biology of Animals Universität Hamburg Hamburg Germany; ^7^ Behavioural Biology, Institute for Neuro‐ and Behavioural Biology (INVB) University of Münster Münster Germany; ^8^ Joint Institute for Individualisation in a Changing Environment (JICE) University of Münster and Bielefeld University Münster Germany; ^9^ Institute for Biochemistry and Biology, Molecular Ecology and Evolution University of Potsdam Potsdam Germany

**Keywords:** anthropogenic disturbance, Cormack‐Jolly‐Seber models, population genetics, red squirrel, *Sciurus vulgaris*, STRUCTURE analysis, urban ecology, urban wildlife

## Abstract

Urbanisation influences ecological and evolutionary processes, including gene flow and survival. Understanding how genetic and demographic rates respond to urbanisation is therefore essential for predicting and managing wildlife viability in human‐dominated landscapes. We tested whether population genetic diversity, genetic structure, and apparent survival of Eurasian red squirrels ( *Sciurus vulgaris* ) vary in concert along an urbanisation gradient. We combined a multi‐year capture–mark–recapture study with microsatellite‐based population genetic analyses at three sites in Berlin, Germany, representing increasing levels of urbanisation. Genetic diversity was broadly comparable among sites, indicating no strong genetic erosion in urban populations. However, weak heterozygote deficits and subtle differences in individual heterozygosity suggest mild restrictions to gene flow along the urban gradient. Bayesian clustering analyses identified two genetic clusters, broadly corresponding to study sites, revealing emerging fine‐scale population structure despite the small spatial extent of the study area. Apparent survival did not differ clearly among sites. Several predictors received similar support, highlighting substantial uncertainty in the drivers of survival in this system. By integrating genetic and demographic data, our study suggests that fine‐scale genetic structuring may emerge before detectable effects on apparent survival become evident. These findings highlight that genetic and demographic responses to urbanisation may occur on different timescales and emphasise the importance of integrated approaches for understanding population responses in human‐modified environments.

## Graphical abstract

Urbanisation can alter both genetic connectivity and demographic performance of wildlife populations, but these responses may occur on different temporal scales. Using multi‐year capture‐mark‐recapture data and microsatellite analyses of Eurasian red squirrels across an urbanisation gradient in Berlin, we found subtle genetic structuring without clear differences in apparent survival. Our results suggest that genetic signals of reduced connectivity may emerge before demographic consequences become detectable.

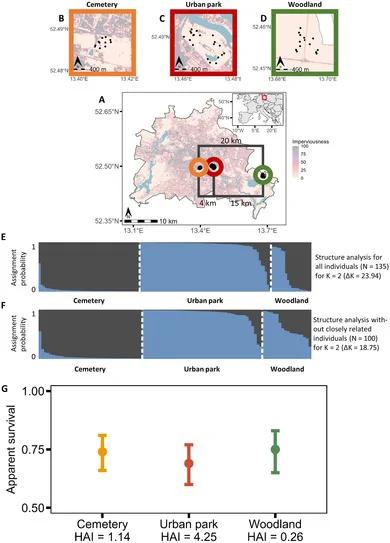

## 1 Introduction

Urbanisation influences ecological and evolutionary processes, including gene flow between populations and their survival, with long‐term consequences for the persistence of wildlife populations in human‐dominated landscapes (Alberti et al. 2020; Mcdonald et al. 2008; McKinney 2002). Understanding how demographic rates and genetic structure change along urban gradients is therefore crucial for urban biodiversity conservation, as genetic erosion or altered survival probabilities may indicate thresholds beyond which populations may struggle to survive and thereby support evidence‐based management decisions (Alberti et al. 2020).

Features of urban habitats can have both constraining and facilitating effects on population dynamics, with outcomes that depend on context and species. These features may involve habitat fragmentation (Kowarik et al. 2025), human disturbances (direct and indirect; Pickett et al. 2001), and altered predator communities (Plaza et al. 2019). Urban habitats also offer access to anthropogenic food sources and novel shelters benefitting numerous species (Ancillotto et al. 2025; Lowry et al. 2013; Sol et al. 2013). Species respond to these features in diverse ways – some thrive, while others decline (Fischer et al. 2015; Santini et al. 2019). Respective responses vary depending on resources or refuge availability (Planillo et al. 2021; Solem et al. 2025) and often involve trade‐offs related to behaviour, space use and diet (Lowry et al. 2013; Rast et al. 2019; Santini et al. 2019). Understanding which factors shape adaptive and demographic responses is crucial for predicting the long‐term persistence of wildlife populations in urban landscapes.

Across and within taxa, the genetic effects of urbanisation are highly variable. Some studies document restricted gene flow and reduced genetic diversity along urban–rural gradients in populations (Hohenlohe et al. 2021; Munshi‐South et al. 2016), whereas others find little differentiation or even elevated heterozygosity, indicating context‐dependence (Miles et al. 2019; Schmidt and Garroway 2021; Ziege et al. 2020). Urban features contributing to these patterns include road barriers, built‐up structures, and the isolation of small habitat patches, or conversely, connectivity facilitated by urban green spaces (Barthel et al. 2020; Stillfried et al. 2017). Beyond effects on genetic structure and diversity, the same urban features also influence demographic processes such as survival (Epps et al. 2005; Fingland et al. 2022; Vandergast et al. 2025). Demographic processes interact with genetic processes such as genetic drift and inbreeding, particularly in small or isolated populations, with potential consequences for individual survival (Hohenlohe et al. 2021; Vandergast et al. 2025). Survival also varies between sexes in many mammalian species, with females often exhibiting longer lifespans than males (Cayuela et al. 2023).

Eurasian red squirrels ( *Sciurus vulgaris* ; hereafter ‘squirrel’) are well‐suited to studying genetic and demographic consequences of urbanisation due to their wide distribution across urban–rural gradients and documented behavioural and ecological flexibility (Drenske et al. 2024; Fingland et al. 2022; Uchida et al. 2019). Habitat structure and level of urbanisation can shape patterns of connectivity among urban populations (Grabow et al. 2022). This raises the question of whether urban landscapes limit gene flow in squirrels, potentially leading to genetically fragmented populations.

Although squirrels have been studied in various regions and contexts (Fingland et al. 2022; Takahata et al. 2024; Tranquillo et al. 2024), including studies about genetics (Rézouki et al. 2014; Selonen et al. 2018; Takahata et al. 2024), integrated assessments of population genetic structure and survival across urban–rural gradients remain scarce. Available urban studies suggest heterogeneous and context‐dependent genetic responses, ranging from reduced diversity and genetic clustering to altered but persistent connectivity (Selonen et al. 2018; Takahata et al. 2024). More broadly, European squirrel populations vary widely in genetic diversity, with some showing high genetic variation (Dozières et al. 2012; Rézouki et al. 2014) and others exhibiting low diversity likely caused by historical bottlenecks or fragmentation (Barratt et al. 1999; Grill et al. 2009; Lucas et al. 2015; Madsen et al. 2015). Despite growing research on urban ecology, the combined effects of habitat fragmentation, survival, and genetic structure remain insufficiently understood (Alberti et al. 2020), particularly for species whose dispersal and population viability depends on habitat connectivity (e.g., squirrels, hedgehogs (Barthel et al. 2020)).

We investigated the population genetics and apparent 6‐month survival rates of squirrels across three study sites spanning an urban–rural gradient from autumn 2021 to spring 2025. We assessed (1) whether population genetic metrics, such as effective allele size and heterozygosity, differ among study sites. We hypothesised reduced genetic diversity and higher inbreeding in urban populations due to strong movement barriers. Further, we hypothesised (2) that there is population genetic structuring among sites. We expected three genetic clusters reflecting restricted gene flow between our study sites. (3) Finally, we assessed whether 6‐month survival of independent individuals varies across study sites and whether any observed genetic differences may explain these patterns. We formulated three alternative predictions regarding survival along the urban–rural gradient. First, we predicted lower survival in more urbanised sites if anthropogenic mortality factors such as habitat fragmentation or traffic dominate. Second, we predicted higher survival in urban sites if positive anthropogenic effects, including supplemental food and human‐mediated care, outweigh mortality risks. Third, we predicted similar survival across study sites if negative and positive urban effects compensate each other. As a result, we hypothesised that genetic differences manifest in functional differences and predicted a positive relationship between survival and individual heterozygosity, and a reduced survival for individuals with high inbreeding. Additionally, we hypothesised males to have a more risky lifestyle and hence expected lower survival in males than in females.

## 2 Materials and Methods

### 2.1 Study Area and Determination of Landscape Characteristics

We conducted the study in Berlin, Germany. Berlin spans 891 km^2^ and is home to nearly 4 million residents (Statistics Office Berlin‐Brandenburg 2025). Green spaces account for 11.6% of the city area and include public parks, cemeteries and other green areas, while forests cover 17.7%. Settlement and transport areas together comprise approximately 59% of the city area (Statistics Office Berlin‐Brandenburg 2024). The city is embedded in a largely forested peri‐urban landscape and is characterised by a moderate continental climate.

To capture variation in urbanisation intensity while ensuring that each site provided suitable habitat for squirrels, we selected three urban green space habitats in Berlin: a woodland, a cemetery, and an urban park, where we captured squirrels from autumn 2021 to spring 2025. The study sites were spatially separated within the city by distances of a few kilometres (Figure 1A). The sites represent increasing levels of urbanisation, defined by differences in habitat type, surrounding landscape, infrastructure, and human activity. To characterise vegetation at each site, we conducted a plant survey in autumn 2022 (for details see S1 including Figure S1, Tables S1 and S2).

**FIGURE 1:**
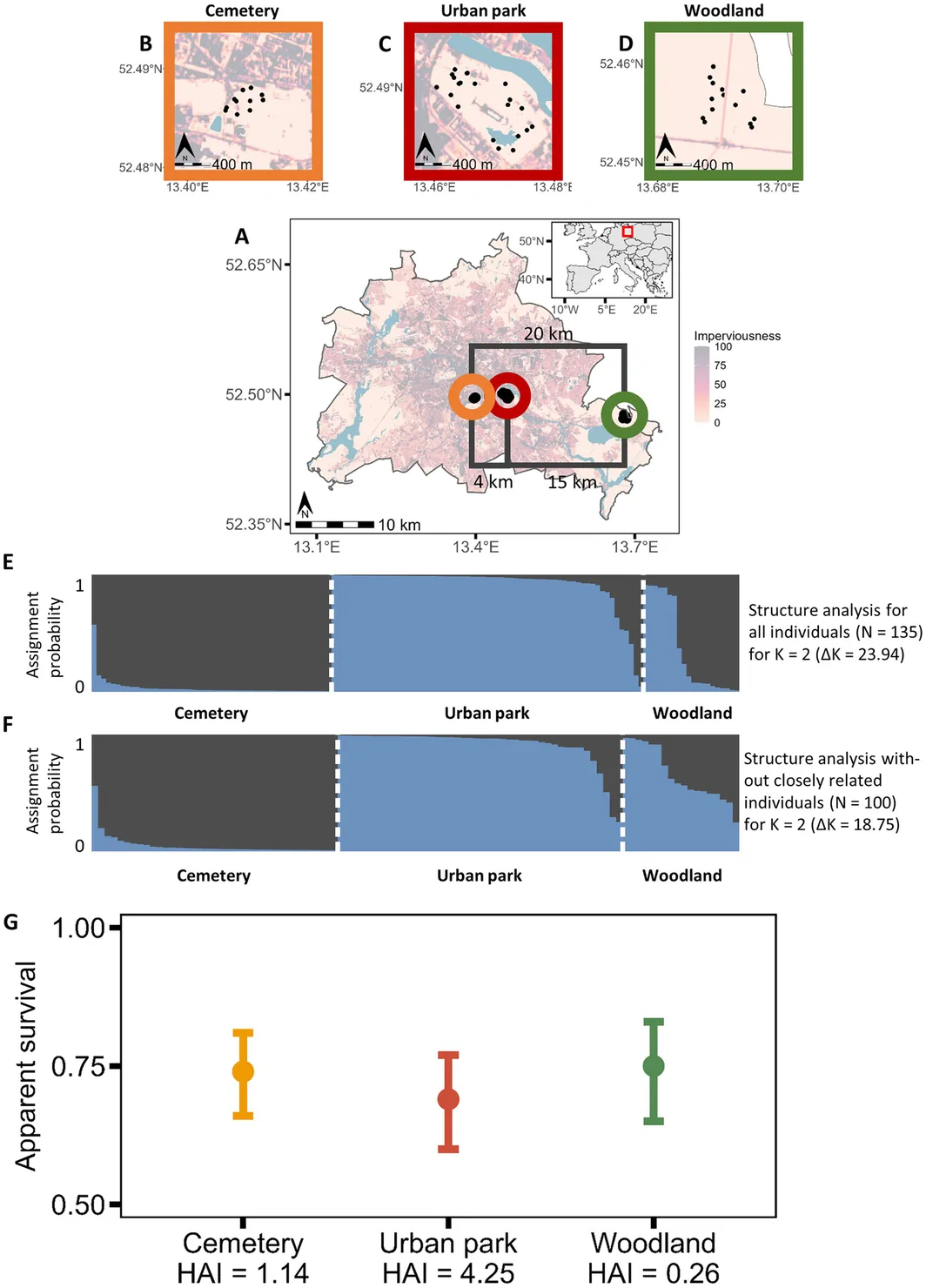
(A) Study areas in Berlin, Germany, with detailed zoom per study area on the cemetery (orange, B), the urban park (red, C) and the woodland (green, D). Black dots represent trap locations. Please note that only 10 traps were active each time, but some traps had to be moved between seasons due to construction work or vandalism, therefore, more dots are visible. (E) Results of the STRUCTURE analysis for all individuals: The most likely number of genetic clusters *K* = 2 was determined with the Evanno method (Evanno et al. 2005). Probabilities of group membership (*Y*‐axis: *Q*‐values) are presented in proportions of two colours (grey, blue) per bar (*X*‐axis). Each bar represents an individual. (F) Results of the STRUCTURE analysis for without closely related individuals: the number of genetic clusters did not change (*K* = 2). (G) Site‐specific estimates of apparent survival in relation to human activity. Points indicate apparent survival estimates, and vertical lines show 95% confidence intervals. HAI values in the *x*‐axis labels indicate the site‐specific Human Activity Index.

To quantify differences in human activity among study sites, we recorded human presence and activity in 30‐min intervals between 07:30 and 12:00 h (woodland: 95 intervals over 26 days; cemetery: 13 intervals over 4 days; urban park: 195 intervals over 26 days). Human activity data for urban park and woodland were collected in autumn 2024, and data for the cemetery in autumn 2025. We counted pedestrians, dogs, bicycles, and other activities. Observations were conducted along streets and footpaths at two locations in the woodland and the cemetery and at four locations in the urban park to account for differences in spatial extent. We standardised activity levels by dividing by eight (i.e., the number of 30‐min intervals per survey period) to obtain a mean number of human activities per 30 min, the human activity index (HAI; see S2 including Figure S2 for details).

The woodland (Figure 1D) near the Berlin‐Brandenburg border represents the low urbanisation level in our study. We captured squirrels within an area of approximately 38 ha embedded in a larger contiguous forest. The site is bisected by a single road with moderate traffic. Human activity is limited to occasional recreation such as walking or hiking, resulting in the lowest HAI of 0.26. Vegetation is characterised by numerous broad‐leaf trees, loose canopy cover, substantial deadwood availability, and ongoing rejuvenation.

The urban cemetery (Figure 1B) covering approximately 10 ha and surrounded by residential areas, parks and other cemeteries, represents the medium level of urbanisation. Human presence is generally low and for short periods of time, resulting in a HAI of 1.14. The site is enclosed by a 1–3 m high stone wall not posing a barrier for squirrels. Vegetation is characterised by old, large‐diameter broad‐leaved trees, low canopy cover, and an absence of deadwood and rejuvenation within vegetation survey plots. Coniferous trees were not recorded on vegetation survey plots; however, European yew ( *Taxus baccata* ) occurs frequently across the cemetery (personal observations).

The urban park (Figure 1C) represents the highest urbanisation level in our study. It covers approximately 96 ha; we captured in approximately 62 ha. It consists of woodland edges surrounding a large central meadow and a small lake used for recreation, dog walking, and events. The park is intersected and bordered by major roads and highly frequented by pedestrians due to its proximity to public transport, resulting in the highest HAI with 4.25. Tree cover consists mainly of broad‐leaved species (mainly *Quercus* and *Acer*), with large average tree diameters, a relatively light canopy cover, and little deadwood. Coniferous trees are absent.

### 2.2 Squirrel Trapping and Sampling

All animal handling followed international, national, and institutional ethical guidelines and was approved by the Berlin State Office for Health and Social Affairs (LaGeSO, permit G0020/21). We applied a standardised capture‐mark‐recapture (CMR) approach to estimate squirrel population sizes across three study sites in Berlin from autumn 2021 to spring 2025.

We captured squirrels biannually in spring and autumn during eight project phases (4 in spring, 4 in autumn) using 10 Tomahawk Live Traps (models 103 and 104, Hazelhurst, USA) per site. Each phase lasted approximately 6 weeks, with each site being visited five times per phase to keep trapping effort constant. We placed traps randomly across forested areas to ensure representative coverage, in suitable vegetation and concealed from paths, either on the ground or at heights of up to 1.5 m. Traps were pre‐baited with walnuts two weeks prior to trapping and refilled every 2–3 days. Camera traps (Seissiger Special‐Cam 3, Anton Seissiger GmbH, Würzburg, Germany) monitored each trap throughout the project phases.

Trapping took place from approximately 07:00–12:00 h, with checks at least every 30 min. Captured squirrels were transferred to a handling cone for safe handling (Koprowski 2002). We marked newly captured squirrels individually with PIT‐tags (EURO I.D., transponder ID 100, Frechen/Köln, Germany) applied subcutaneously in the shoulder region and identified recaptured individuals based on their PIT‐tag. During each capture and recapture, we took, among other data, standard morphometric measurements (body, tail and hind‐foot length, and body mass), sex, and physical condition. An ear tissue sample (2 mm^2^) for genotyping was collected once per individual. All handling lasted 15–20 min before release at site of capture. Samples were stored at −20°C and later transferred to −80°C (for details see S3.1, Figure S3).

### 2.3 Estimating Genetic Diversity

DNA extraction, genotyping and genotyping error estimation followed standard procedures (e.g., Stillfried et al. 2017; for details see S4.1, S.4.2 with Table S3). To assess genetic diversity within and among study sites we calculated standard population genetic metrics per population and averaged across loci. Within each site we estimated the total and mean number of alleles (*N*_*A*_), Allelic richness (*A*_*R*_), Allele frequencies (*A*_*F*_), number of private alleles (PA), observed (*H*_*O*_) and expected heterozygosity (*H*_*E*_) with the Weir & Cockerham approach, the standardised individual multilocus heterozygosity (sMLH), the proportion of homo‐ and heterozygous individuals per locus, effective number of alleles (*A*_*E*_), individual inbreeding coefficient (*F*) and the inbreeding of individuals relative to their subpopulation (*F*_IS_). For the analysis of genetic diversity we used R v.4.4.2 (R Core Team 2024) with the user interface RStudio 2024.09.1+394 (Posit team 2024) and the packages adegenet v.2.1.11 (Jombart 2008; Jombart and Ahmed 2011), hierfstat v.0.5‐11 (Goudet and Jombart 2022), inbreedR v.0.3.3 (Stoffel et al. 2016), pegas v.1.3 (Paradis 2010) and poppr v.2.9.6 (Kamvar et al. 2014, 2015).

### 2.4 Estimating Population Genetic Structure

We analysed the squirrel population genetic structure using a Bayesian genetic clustering approach with STRUCTURE v.2.3.4 (Pritchard et al. 2000). To infer the number of ancestral genetic clusters (*K*), we ran 25 independent runs for *K* = 1–10, using an admixture model with correlated allele frequencies and a burn‐in of 10,000 followed by 100,000 MCMC iterations. We inferred Alpha, the dirichlet parameter for degree of admixture, and lambda, a parameter for allele frequency distribution. We determined the most likely *K* based on the log‐likelihood values and their convergence associated with each *K* by using the Δ*K* method (Evanno et al. 2005). We applied this method through the pophelper package (Francis 2017). Afterwards we calculated the individual percentages of cluster membership (*q*) by averaging *q* over the 25 runs for the best *K*.

To disentangle population structure and family structure (Rodríguez‐Ramilo and Wang 2012), we calculated pairwise relatedness between individuals per study site using the Queller and Goodnight (1989) kinship estimator implemented in the R package related v.1.0 (Pew et al. 2015). Pairs with a kinship coefficient greater than 0.5 were considered close relatives; in such cases, we removed the individual that showed higher relatedness to multiple others. Subsequently, we reran the structure analysis to assess whether the previously detected clusters represented family groups or broader population‐level clusters.

### 2.5 Survival Analyses

To estimate apparent 6‐month survival (*Φ*; from project phase to project phase) and detection probabilities (*p*), we used Cormack‐Jolly‐Seber models (CJS; Cooch and White 2025) with R package RMark v.3.0.0 (Laake 2013). We defined a capturing event as the capture of an individual during one of the designated project phases of this study. We included all trapped individuals irrespective of age, as only squirrels capable of independent movement and foraging were captured; precise age determination in this species is unreliable. We set *p* to 0 for the cemetery during project phases 1 and 2 and for the woodland during project Phase 4, as no sampling took place at these sites during these periods.

Because levels of urbanisation and trapping effort differed among study sites, we included ‘study site’ as a grouping variable. We included ‘sex’ as second grouping variable, and individuals with unknown sex were excluded from the analyses (*n* = 3). First, we modelled *p* while keeping *Φ* constant (~1). In this step, we evaluated models with constant *p* (~1), as well as models including study site (woodland, cemetery, urban park), sex (F, M), trapping effort (measured as trapping hours per study site and project phase; time dependent), and season (spring, autumn; time dependent, corresponding to the 6‐month survival interval). We tested single‐predictor models, additive combinations, and biologically relevant two‐way interactions (for details see S5.1). We assumed that squirrels in our urban areas are more likely to be detected due to their familiarity with human presence. We assumed differences between sexes and seasons, as females may be less detectable than males during spring, likely due to increased maternal care. Furthermore, we assumed that *p* depends on trapping effort, requiring a correction factor to account for this and we assumed these factors to influence each other. We performed model selection using the corrected Akaike's information criterion for critical sample sizes (AICc). Models with ΔAICc < 2 were considered to have comparable support (Burnham and Anderson 2004). We used an information‐theoretic approach and compared a priori candidate models based on our hypotheses using AICc. We used the best‐supported detection model as the detection component for the subsequent survival analyses.

In the second step, we modelled *Φ* while fixing *p* to the best‐supported structure from step one. Here, we evaluated study site, sex, season, standardised individual multilocus heterozygosity (sMLH), individual inbreeding coefficient (*F*), and human activity index (HAI) per 30‐min interval as predictors. We used sMLH and *F* as individual‐level genetic metrics to assess potential effects of genetic diversity and inbreeding on *Φ*. Besides the genotyped squirrels, six additional individuals without genetic samples were included in the survival analysis. Here, we used site‐level mean values for *F* and sMLH. As before, we tested models with single predictors, additive combinations, and biologically meaningful interactions. We included a constant survival model and four full models, each incorporating one of the genetic indices, with and without an interaction term, for comparison. From this selection of models, we chose the one with the lowest AICc.

## 3 Results

We recorded 540 capture events (woodland: 85, cemetery: 201, urban park: 254, Figures S4 and S5) across all study areas over eight project phases, corresponding to 141 individuals (Figure S6). Sex ratio was approximately balanced overall with 67 females and 71 males and at each study site, with males only slightly more common than females (Figure S7). Trapping success differed among sites, with 21 in the woodland (8 f, 13 m), 53 in the cemetery (25 f, 27 m, 1 unknown), and 67 individuals captured in the urban park (34 f, 31 m, 2 u). Of all captured individuals, 93 squirrels were recaptured at least once (range 1–18), corresponding to 66% of the sampled population (71% woodland, 66% cemetery, 64% urban park). We captured individuals on average 3.83 ± 3.66 times (range: 1–19) and on average in 1.94 ± 1.24 project phases (range: 1–7).

### 3.1 Genetic Analyses

We obtained genetic samples from 158 squirrels (Figure S8). After accounting for duplicates and genotyping errors, we identified 135 unique individual genotypes: 20 from the woodland (8 f, 12 m), 50 from the cemetery (24 f, 25 m, 1 u) and 65 from the urban park (34 f, 30 m, 1 u). Three individuals had missing data at three loci (threshold for inclusion was two loci). Two individuals were from the urban park and one individual (ID 7F24D77) from the woodland, from which we had the lowest number of captured individuals (*n* = 21). We did not detect consistent linkage disequilibrium among loci. We therefore analysed three datasets: all individuals (*n* = 135), a reduced dataset (*n* = 132) and a second reduced dataset including individual 7F24D77 (*n* = 133), each based on 16 loci. As the results were very similar, all following results are based on the complete data set. Only three out of the 16 loci (Rsu5, Scv14, Scv31) had a somewhat elevated probability for the presence of null alleles (< 0.2; Table S4). These loci also deviated from the HWE.

#### 3.1.1 Genetic Diversity

Across the three study sites, squirrel populations exhibited similar genetic diversity, with comparable values for *N*_*A*_, mean *N*_*A*_, *A*_*E*_ and *A*_*R*_ (Table 1; S4.3, Table S4). While some diversity measures were numerically higher in the woodland population and lower in the cemetery population, differences among sites were generally small. Each population, however, also harboured several private alleles: 15 in the woodland population, 6 in the cemetery population and 11 in the urban park population. *H*_*O*_ and *H*_*E*_ were generally similar across sites, although the cemetery population showed a slight deficit of heterozygotes (paired *t*‐test, *t* = −2.20, df = 15, *p* = 0.044). *F*_IS_ suggested low to moderate heterozygote deficits across sites (Table 1), which may reflect mild inbreeding, interrupted gene flow and the beginning of segregation in urban sites. We detected significant sMLH differences among populations (Kruskal–Wallis test: *χ* ^2^ = 6.55, df = 2, *p* = 0.038), with post hoc Dunn tests revealing lower sMLH in the urban park population than in the woodland population (adj. *p* = 0.028). Overall, the results indicated that urbanisation does not drastically reduce genetic diversity in squirrels, but subtle patterns of heterozygote deficit and private alleles suggest localised structuring.

**TABLE 1:** Descriptive population genetics.

Site	*N*	*N*_*A*_	Mean *N*_*A*_ (SD)	*A*_*E*_ (SD)	*A*_*R*_ (SD)	*H*_*e*_ (SD)	*H*_*o*_ (SD)	sMLH (SD)	*F* (SD)	*F*_IS_ (CI)	PA
Woodland	20	84	5.25 (2.32)	3.33 (1.77)	5.22 (2.32)	0.64 (0.14)	0.60 (0.17)	1.12 (0.25)	0.19 (0.11)	0.05 (−0.03, 0.13)	15
Cemetery	50	76	4.75 (2.21)	2.87 (1.13)	4.33 (1.78)	0.60 (0.14)	0.53 (0.16)	1.00 (0.28)	0.24 (0.12)	0.11 (0.03, 0.19)	6
Urban park	65	80	5.00 (2.78)	3.09 (2.06)	4.36 (2.11)	0.56 (0.22)	0.52 (0.21)	0.96 (0.23)	0.22 (0.11)	0.09 (0.02, 0.18)	12

*Note:* Values are presented as mean (SD) for all parameters except FIS, for which 95% confidence intervals are shown.

Abbreviations: *A*_*E*_, effective number of alleles; *A*_*R*_, allelic richness; CI, 95% confidence interval; *F*, individual inbreeding coefficient; *F*_IS_, inbreeding of individuals relative to their subpopulation; *H*_*E*_, expected heterozygosity; *H*_*O*_, observed heterozygosity; mean *N*_*A*_, mean number of alleles across all loci; *N*, number of individuals; *N*_*A*_, total number of alleles across all loci; PA, private alleles; SD, standard deviation; sMLH, standardised multilocus heterozygosity.

#### 3.1.2 Population Genetic Structure

STRUCTURE analysis identified two moderately strong genetic clusters in the squirrel metapopulation (mean LnP(*K*) = −5208.87, Δ*K* = 23.94; Table S5 and Figure S9). These clusters corresponded only partly to the study sites: individuals from the woodland and the cemetery mainly belonged to Cluster 1, whereas most individuals from the urban park grouped in Cluster 2 (Figure 1E), despite the cemetery being less than 4 km away. Assignment of squirrels from both the woodland and the cemetery to the same cluster was unexpected because both sites are 20 km apart from each other. Based on geographic distance alone, we would have expected the woodland population to form a distinct cluster, with the two urban sites either clustering together or forming separate clusters. However, this pattern was not observed. Pairwise F_ST_ estimates showed a similar pattern and did not indicate stronger genetic differentiation among study sites (Table S7).

To avoid clustering being dominated by an underlying family structure, we removed 35 highly related individuals (distributed across sites: woodland = 2 of 20 individuals, 10%, cemetery = 12 of 50, 24%, urban park = 21 of 65, 32%), and reanalysed the data. A structure of two clusters still remained the best representation for the allele distribution. However, the cluster representation was weaker than before (mean LnP(*K*) = −4024.64, Δ*K* = 18.75; Table S6 and Figure S10), and the woodland population had a larger proportion of admixed individuals (Figure 1F).

### 3.2 Survival Analyses

The best‐supported model for detection included sex and season as additive effects (AICc = 463.35, weight = 0.31, Table S8). Four out of 14 models tested had ΔAICc < 2; all of these included sex as a predictor and three additionally included season. Males were detected with a higher probability, and squirrels were more likely to be detected and recaptured in autumn.

We compared 20 CJS‐models to investigate factors influencing survival (*Φ*) of squirrels (Table S9). Five models had ΔAICc < 2 and were considered competitive. Overall, support for individual predictors was weak, as several models received similar support, including the null model. The model including inbreeding coefficient *F* was the best‐supported model when combined with a detection dependent on sex and season (*Φ* ~ *F*, *p* ~ sex + season; AICc = 463.11, weight = 0.20). However, this model was essentially identical to the null model with constant survival (AICc = 463.35, weight = 0.18). Models including sex, sMLH, study site, and HAI (Figure 1G) received similar support (ΔAICc < 2), suggesting that no single covariate clearly outperformed alternative explanations. Parameter estimates were associated with considerable uncertainty, with confidence intervals broadly overlapping among predictor values. *F* was not significant, that is, the CI overlapped 0, but there was a tendency to higher survival with increased inbreeding (Table 2; *β* = 2.19 ± 1.51; Figure S11 and Table S10). Differences in survival probability between the lowest and highest measured *F*‐value were 0.2.

**TABLE 2:** Survival probabilities (inverse logit) of the best supported survival models with ΔAICc < 2.

Survival predictor	Detection probability predictor	Range of predictor variables (value); predicted apparent survival probability (95% CI)	AICc	ΔAICc
*F*	Season + Sex	Min F (0.09): 0.66 (0.55–0.76) Max F (0.62): 0.86 (0.64–0.96)	463.11	0.00
Intercept (mean survival); null model		0.72 (0.65–0.78)	463.35	0.24
Sex		Male: 0.69 (0.60–0.77) Female: 0.75 (0.65–0.83)	464.53	1.42
sMLH		Min sMLH (0.35): 0.78 (0.61–0.89) Max sMLH (1.63): 0.65 (0.47–0.80)	464.70	1.58
Human activity		Woodland (low HAI 0.26): 0.75 (0.65–0.83) Cemetery (low to medium HAI 1.14): 0.74 (0.66–0.81) Urban park (high HAI 4.25): 0.69 (0.60–0.77)	464.71	1.60

Abbreviations: CI = confidence interval; *F* = individual inbreeding coefficient; sMLH = standardised multilocus heterozygosity.

## 4 Discussion

Our study indicates subtle genetic differentiation among urban squirrel populations in Berlin, detectable even at a small spatial scale relative to the dispersal distance of squirrels. Overall genetic diversity was comparable across study sites, while fine‐scale metrics pointed to fine constraints on gene flow towards more urbanised areas. These genetic patterns were accompanied by marginal differences in survival.

### 4.1 Genetic Diversity

We found no evidence for reduced genetic diversity in urban populations relative to the woodland (Table 1). Nevertheless, subtle differences emerged at the individual level: low to moderate F_IS_ across sites, weak but significant heterozygote deficits in the cemetery population, and a lower sMLH in the urban park compared to the woodland. These patterns emerged despite the smaller sample size in the woodland population, suggesting that higher diversity is unlikely to be an artefact of sampling effort alone. These patterns suggest early‐stage genetic structuring associated with reduced connectivity, like founder effects, without evidence for advanced inbreeding or genetic erosion. The absence of reduced genetic diversity despite evidence of genetic structuring may indicate that current levels of connectivity remain sufficient to maintain genetic diversity, even if movement among populations is partially restricted. This suggests that local landscape context, including movement barriers such as major waterways and infrastructure (Munshi‐South et al. 2016), may be more important than urbanisation per se.

Despite urban–rural differences, genetic diversity in our populations was high relative to other squirrel populations. Mean A_R_ and heterozygosity exceeded values reported for several squirrel populations in France, Great Britain, and on Jersey (Hardouin et al. 2021; Ogden et al. 2006; Rézouki et al. 2014; Simpson et al. 2013), and were similar to values determined in populations from southern Germany, Italy, and Finland (Hardouin et al. 2021; Selonen et al. 2018; Trizio et al. 2005). Although many of these studies report population‐wide rather than site‐specific estimates, the comparison indicates that squirrels in Berlin retain substantial genetic variation despite potential barriers associated with urban infrastructure, like major roads with often four to eight lanes, railway corridors, and waterways. Takahata et al. (2024) found reduced nucleotide diversity in urban populations in Japan compared to peri‐urban and rural sites. While we observed fewer significant differences using microsatellite markers, subtle heterozygote deficits and population structuring in urban sites similarly suggest that urbanisation may influence population genetic patterns, although the magnitude of these effects was considerably weaker than those reported by Takahata et al. (2024). Inbreeding levels in our study populations were also low and comparable to other European populations (Rézouki et al. 2014).

Across and within taxa, evidence for urban effects on genetic diversity is mixed, with reduced diversity reported for some species (Stillfried et al. 2017; Vargová et al. 2023) but high diversity maintained in others (Desvars‐Larrive et al. 2017; Mihalik et al. 2025; Ziege et al. 2020). Comparative studies indicate that such patterns depend strongly on species traits, landscape configuration, and how urbanisation is quantified (Miles et al. 2019; Schmidt and Garroway 2021).

Differences in effective sampling area and site‐specific settings (e.g., vegetation, habituation to humans) may have influenced capture success and should be considered when comparing sample sizes and population patterns among sites. The woodland site represents only a small section of a much larger continuous forest, providing direct access to extensive surrounding habitat beyond the effective sampling area. In contrast, the cemetery and urban park are embedded within a fragmented urban matrix characterised by major roads, railway infrastructure and built‐up areas. Squirrel densities are generally higher in urban parks than in near‐natural forests (Babińska‐Werka and Żółw 2008), which, together with the larger sampled area, may partly explain the higher number of captures in the urban site.

While small reductions in genetic diversity may not have immediate demographic consequences, they could constrain adaptive potential under ongoing environmental change (Schmidt et al. 2020). According to Schmidt et al. (2024), emerging genomic approaches offer powerful opportunities to disentangle neutral from adaptive genetic variation and to evaluate long‐term evolutionary consequences of urbanisation in wildlife populations.

### 4.2 Population Genetic Structure

Across all STRUCTURE analyses, we consistently identified two genetic clusters, irrespective of whether highly related individuals were included, leading us to reject our hypothesis of three distinct genetic clusters. Despite the limited spatial extent of the study area, clustering broadly aligned with the urban landscape configuration. Whereas both clusters were almost evenly distributed in the woodland, both urban sites were either dominated by one or the other cluster. Although this pattern did not correspond to geographic distance alone, it may reflect differences in landscape context and connectivity as well as founder effects, with the woodland population showing the highest admixture and urban populations exhibiting more distinct genetic signatures. The high levels of admixture in the woodland are consistent with greater connectivity and potential influx from surrounding rural areas.

Previous connectivity modelling by Grabow et al. (2022) identified several potential habitat corridors within the urban landscape. The urban park investigated in the present study was identified as an important node of this network between woodland area and the cemetery. Interestingly, while woodland has an even share of genetic clusters, the urban park and cemetery have uneven shares, pointing to founder effects at both urban sites. However, the connectivity predictions were derived from habitat suitability models based on citizen science observations and therefore represent potential rather than demonstrated movement pathways. Consequently, the subtle genetic structuring observed in our study may indicate that the presence of suitable habitat corridors does not necessarily translate into unrestricted gene flow and that urban infrastructure may still influence functional connectivity among populations. Our genetic results therefore provide an independent line of evidence that complements habitat‐based connectivity predictions and may help assess how well potential corridors facilitate gene flow in urban landscapes.

The low ΔK values supporting two clusters likely reflect the limited spatial scale of the study. STRUCTURE analyses can favour two clusters (Janes et al. 2017; Kalinowski 2011), but complementary genetic metrics, including low to moderate F_IS_, were consistent with weak population structuring. Together, these lines of evidence suggest that subtle genetic differentiation can emerge in urban environments even while overall connectivity is largely maintained. Although the patterns indicate reduced gene flow across the urban matrix, there is no evidence for strong inbreeding or advanced genetic isolation. Rather than reflecting long‐established population divergence, the observed clustering likely represents an early stage of genetic structuring associated with urban barriers.

Within this context of reduced gene flow, differentiation appears to be driven by site‐specific processes rather than city‐wide isolation. These patterns are consistent with a scenario in which admixed individuals from the woodland population colonised the urban sites, and subtle initial differences in cluster membership were subsequently accentuated under limited gene flow, leading to the predominance of different genetic backgrounds in the park and cemetery. Stronger functional barriers surrounding the urban park, such as high‐traffic transport infrastructure, may further reinforce this pattern. In this context, we found marginally lower survival probabilities in the highly disturbed urban park in relation to the two other locations. While the urban park may represent a site with subtle genetic differentiation rather than a fully isolated unit, the clustering detected here might reflect fine‐scale differentiation within our study sites. With broader sampling across Berlin, and particularly across Germany, the observed clusters would likely represent only a small component of wider regional genetic variation.

Importantly, the genetic structure observed in our study was robust to the removal of closely related individuals, indicating that clustering was not driven by family structure. This contrasts with findings from urban hedgehog populations in Berlin, where apparent genetic clustering disappeared after accounting for relatedness (Barthel et al. 2020). Similar weak to moderate genetic structuring has been reported for wild boar populations in Berlin (Stillfried et al. 2017). In contrast, several highly urban‐adapted species show little or no population genetic structure, including birds and rodents, likely due to high mobility or large effective population sizes that buffer against genetic drift (Desvars‐Larrive et al. 2017; Mihalik et al. 2025). Similar context‐dependent patterns occur in other vertebrates, with some urban populations retaining high genetic diversity despite subtle population subdivision, whereas others show reduced diversity alongside weak clustering (Vargová et al. 2023; Ziege et al. 2020).

Similar context‐dependent patterns have been reported for squirrels in other urban contexts. Takahata et al. (2024) identified two genetic clusters in the Obihiro region (Japan), with stronger differentiation among urban sites, indicating restricted gene flow relative to rural areas. In contrast, Selonen et al. (2018) found no urban–rural differentiation in Finnish squirrel populations in Turku but reported differentiation among urban populations. These contrasting results highlight how scale and structural complexity shape genetic outcomes: Berlin is a large, densely populated metropolis with extensive infrastructure and fragmented green spaces, whereas Turku and Obihiro are smaller, less densely urbanised cities.

### 4.3 Survival

Overall, we found no clear evidence for differences in apparent survival among study sites. While the model including F had the lowest AICc and hence explained parts of the variance, several competing models received similar support (ΔAICc < 2), including the null model, and no single predictor clearly dominated. Neither study site, sex, sMLH, nor *F* showed clear support as predictors of apparent survival, and confidence intervals were generally broad. This model uncertainty likely reflects limited statistical power associated with the relatively short study period and moderate sample sizes. Moreover, estimated survival represents apparent 6‐month survival and cannot distinguish mortality from permanent emigration (Cooch and White 2025).

Despite this uncertainty, apparent survival estimates were broadly similar across study sites (0.69–0.75, Table 2). Survival was lowest in the urban park, where predicted 6‐month survival was approximately 6% lower than at the other sites, suggesting a possible tendency towards demographic costs under high anthropogenic pressure. If maintained over multiple years, even relatively small differences in apparent survival could potentially accumulate, although substantial uncertainty remains. Although not strongly supported, males tended to show slightly lower survival, consistent with sex‐specific life‐history strategies.

Contrary to our prediction, neither sMLH nor *F* showed clear support as predictors of survival, and effect directions were inconsistent with classical heterozygosity‐fitness expectations. Interestingly, the model including *F* suggested a weak, non‐significant positive association with apparent survival, contrasting with the classical expectation of reduced survival under higher inbreeding. In the absence of strong genetic differentiation or inbreeding depression, subtle differences in genetic diversity are not necessarily expected to translate directly into detectable differences in apparent survival. Genetic structure can arise through several processes, including founder effects, genetic drift, non‐random recruitment or local population turnover, without necessarily being associated with reduced apparent survival. Thus, the absence of detectable survival differences in our study does not imply that demographic effects will inevitably emerge at a later stage. Rather, the distinct genetic signature of the urban park population, despite evidence for connectivity with the surrounding landscape, suggests that local population processes have shaped this site. Uneven recording of human activity among sites may have limited the ability of the human activity index to capture effects on apparent survival. In addition, apparent survival in urban wildlife populations is likely influenced by multiple interacting ecological factors that were not explicitly quantified in this study, including variation in resource availability, predation pressure, disease, and other environmental conditions. Future studies integrating a broader range of ecological variables may help explain the remaining variation in survival among individuals and populations.

Different studies indicate that genetic effects on survival are highly context dependent. In Apennine brown bears (*Ursus arctos marsicanus*), reduced genome‐wide diversity and elevated inbreeding did not reduce population persistence, likely due to selection maintaining variation at loci associated with survival and behaviour (Fabbri et al. 2025). In contrast, positive heterozygosity‐fitness relationships have been reported in fragmented populations of cactus wrens ( *Campylorhynchus brunneicapillus* ), where multilocus heterozygosity was associated with higher survival (Vandergast et al. 2025). Other studies report weak or conditional effects of genetic diversity on survival across taxa, including mammals, birds and amphibians, suggesting that demographic and environmental context can mask or modulate genetic effects (Annavi et al. 2014; Canal et al. 2014; Pröhl and Rodríguez 2023). Supplemental feeding can further modify survival patterns by increasing local densities at feeding sites (Wist and Dausmann 2024) and was observed to a high degree at the cemetery, while potentially elevating disease risks (Shuttleworth et al. 2015).

Finally, genetic diversity does not necessarily predict survival. As emphasised by Lowe and Allendorf (2010), genetic connectivity does not equate to demographic connectivity. Integrating genetic data with demographic analyses is essential for understanding population persistence in human‐modified landscapes (Hohenlohe et al. 2021). In our study, integrating genetic and demographic data did not reveal strong links between genetic structure and apparent survival, suggesting that genetic effects on short‐term survival may be weak, context dependent, or detectable only at broader temporal or spatial scales.

## 5 Conclusions

Assessing genetic structure and survival within the same urban populations allowed us to contrast genetic and demographic responses to urbanisation, revealing that fine‐scale genetic structure can emerge without immediate consequences for apparent survival. Despite subtle genetic differentiation and indications of reduced connectivity towards more urbanised sites, overall genetic diversity remained high and was not associated with strong differences in apparent survival.

These findings suggest that certain urban landscape configurations may sustain genetically and demographically stable populations, at least over short temporal scales. Green spaces and tree‐covered areas may facilitate movement and gene flow, buffering short‐term survival despite emerging genetic structure. At the same time, subtle constraints on connectivity highlight that early genetic structuring can precede demographic effects. Combining genetic data with fitness proxies such as survival (Hohenlohe et al. 2021) therefore provides a more nuanced assessment of population persistence and suggests that short‐term demographic stability does not preclude longer‐term vulnerability if connectivity declines further.

## Author Contributions

**Sinah Drenske:** conceptualization (lead), data curation (equal), formal analysis (lead), funding acquisition (equal), investigation (lead), methodology (equal), project administration (equal), validation (lead), visualization (lead), writing – original draft (lead), writing – review and editing (lead). **Conny Landgraf:** conceptualization (lead), investigation (equal), project administration (equal). **Alina Theresa Berger:** formal analysis (supporting). **Alina Doreen Stemmer:** formal analysis (supporting), investigation (supporting). **Aimara Planillo:** formal analysis (supporting), methodology (equal), writing – review and editing (equal). **Johanna Leona Eul:** investigation (equal), methodology (equal). **Bianca Wist:** methodology (equal), writing – review and editing (equal). **Kathleen Röllig:** conceptualization (equal), investigation (equal), writing – review and editing (equal). **Ashlee Jean Mikkelsen:** formal analysis (equal), methodology (equal), writing – review and editing (equal). **Melanie Dammhahn:** conceptualization (equal), investigation (supporting), methodology (equal), writing – review and editing (equal). **Jörns Fickel:** data curation (equal), formal analysis (equal), methodology (equal), writing – review and editing (equal). **Stephanie Kramer‐Schadt:** conceptualization (lead), data curation (equal), funding acquisition (equal), investigation (equal), methodology (equal), project administration (equal), resources (lead), supervision (lead), writing – review and editing (equal).

## Funding

S.D. received funding from the Elsa‐Neumann foundation and Technische Universität Berlin. S.D. and S.K.‐S. were associated with the DFG Research Training Group ‘BioMove’ (DFG‐GRK 2118/2).

## Disclosure

*Statement on inclusion*: This study was conducted in Berlin, Germany, and the majority of the author team is based in Germany, including researchers working at the IZW in Berlin. All authors contributed to the study through involvement in the study design, data collection, data interpretation, and/or manuscript development, with additional collaboration from colleagues at other institutions. Fieldwork was carried out with the appropriate permits (LaGeSO, permit G0020/21) and in coordination with local authorities and site managers responsible for the study areas. Wherever relevant, we considered literature from the study region, including German‐language sources for contextual information such as land use and site characteristics. The study is embedded in the local ecological and urban context and aims to contribute to both fundamental research and the understanding of wildlife populations in highly urbanised environments. In addition, study outcomes are shared through public outreach activities, including public presentations.

## Conflicts of Interest

The authors declare no conflicts of interest.

## Supporting information


**Table S1:** Feeding plants of the Eurasian red squirrel ( *Sciurus vulgaris* ).
**Figure S1:** (A) Distribution of the NMDS (grey dots) with influence of the response variables (blue dots and arrows) distinguished between *R* ^2^ values below 0.5 (lower influence, light blue arrows) and *R* ^2^ values above 0.5 (higher influence, dark blue arrows). The following abbreviations were used for the response variables: ‘FPA’ for feeding plant amount, ‘av_DBH’ for average DBH in m, ‘av_BA’ for average basal area in m^2^, ‘q0–q2’ for the Hill numbers q0–q2, ‘Nr_Con’ for number of conifers and ‘Nr_BLT’ for number of broad‐leaf trees. ‘Vit_lvl’ indicates the average vitality level per plot, ‘CC’ the canopy closure and ‘D_s’ stands for the deadwood share in addition to ‘Rejuv’ depicting the rejuvenation in percentage. (B) NMDS of vegetation characteristics with depicted explanatory variables ‘Locations’ and ‘Distances to path’.
**Table S2:** Results of the environmental fitting analysis of the response variables from the NMDS.
**Figure S2:** Human activity index (HAI) per study site.
**Figure S3:** Details of the capture and handling procedure. (A) Squirrel in a Tomahawk live trap. (B) Individual placed in a handling bag during weighing using a spring scale. (C) Identification of recaptured individuals using a PIT tag reader. (D) Collection of an oral swab. (E) Sex determination; the individual shown is male. (F) Release of the squirrel at the capture site. Pictures by Jon A. Juárez.
**Figure S4:** Capturing events of squirrels per study site.
**Figure S5:** Relationship between weight (*y*‐axis) and body length (*x*‐axis) of squirrels across the study sites.
**Figure S6:** Number of captured individuals per study site.
**Figure S7:** Sex ratio of captured squirrels per study site.
**Figure S8:** Number of tissue samples for the genetic analysis per study site.
**Table S3:** Properties of the microsatellite loci used in this study. Multiplex: indicates in which multiplex reaction the primer pair was used. *N* = number of samples successfully analysed, in total we have 158 samples. F = forward primer; R = reverse primer.
**Table S4:** Locus‐level genetic diversity measures. Null = oosterhout statistic for null alleles; *N*_*A*_ = number of alleles; *A*_*R*_ = allelic richness; PA = private alleles; *H*_*O*_ = observed heterozygosity; *H*_*E*_ = expected heterozygosity; *A*_*E*_ = Effective number of alleles; *F*_IS_ = inbreeding coefficient; U = urban park; C = cemetery; W = woodland area; T = Total.
**Table S5:** Result table of the Cluster analysis with all individuals (*N* = 135). MEAN LN PROB = mean estimated ln probability of data. SD mean LN prob. = standard deviation. Δ*K* = third derivate; LNK1 = first derivate; LNK2 = Second derivate.
**Figure S9:** Determination of the most likely number of genetic clusters (*K*) using the Evanno method (Evanno et al. 2005) based on STRUCTURE analyses for all individuals (*N* = 135). (A) Mean estimated ln probability of data ± SD. (B) First derivate ± SD. (C) Second derivate ± SD. (D) Δ*K*. On the *x*‐axis you can always see the number of clusters (*K*).
**Table S6:** Result table of the Cluster analysis without closely related individuals (*N* = 100). MEAN LN PROB = mean estimated ln probability of data. SD mean LN prob. = standard deviation. Δ*K* = third derivate; LNK1 = first derivate; LNK2 = second derivate.
**Figure S10:** Determination of the most likely number of genetic clusters (*K*) using the Evanno method (Evanno et al. 2005) based on STRUCTURE analyses excluding closely related individuals (*N* = 100). (A) Mean estimated ln probability of data ± SD. (B) First derivate ± SD. (C) Second derivate ± SD. (D) Δ*K*. On the *x*‐axis you can always see the number of clusters (*K*).
**Table S7:** Above the diagonal: Pairwise *F*_ST_ values with Confidence Interval (CI). Below the diagonal: distance in km between the study sites.
**Table S8:** Model list for the identification of the best predictors for detection probability.
**Table S9:** Model list for the identification of the best predictors for survival.
**Figure S11:** Apparent survival probability in relation to the individual inbreeding coefficient *F*.
**Table S10:** Model estimates on the logit scale of the best squirrel survival model. Study sites were denoted as groups. CI = confidence interval; SE = standard error. The intercept represents the following levels of the factor variables: Season = Autumn; Sex = females.

## Data Availability

The data and scripts can be found in the Github repository of the Department of Ecological Dynamics of the Leibniz Institute for Zoo and Wildlife Research: https://github.com/EcoDynIZW/Drenske_2026_EcolEvol and are permanently archived on Zenodo at https://doi.org/10.5281/zenodo.22750358.
