# Supplementary material for: Urbanisation Leaves a Genetic Signature but No Detectable Demographic Effects in a Small Mammal Population
Source: Ecol Evol. 2026 Oct 5;16(10):e74273. doi: 10.1002/ece3.74273 (PMC13639385; doi:10.1002/ece3.74273)
Supplement: Supplementary file 1 [file ECE3-16-e74273-s001.docx]

Supporting Information

For the manuscript with the title:
Decoupled genetic and demographic responses to urbanization in a small mammal population

Table of Content

[S1: Vegetation survey	2](#_Toc221806635)

[S1.1 Methods	2](#_Toc221806636)

[S1.2 Results	3](#_Toc221806637)

[S2: Human activity index (HAI)	5](#_Toc221806638)

[S3: CMR	6](#_Toc221806639)

[S3.1 Detailed description of the CMR-process	6](#_Toc221806640)

[S3.2 Results of capturing and sampling	8](#_Toc221806641)

[S4: Genetic analyses	11](#_Toc221806642)

[S4.1 DNA extraction, genotyping and genotyping error estimation	11](#_Toc221806643)

[S4.2 Information on microsatellite markers	12](#_Toc221806644)

[S4.3 Results of genetic analysis per locus	13](#_Toc221806645)

[S4.4: Results of population genetic structure analysis	15](#_Toc221806646)

[S4.5: Discussion: Comparison with other squirrel studies	17](#_Toc221806647)

[S5: Survival analysis	18](#_Toc221806648)

[S5.1 Detailed methods	18](#_Toc221806649)

[S5.2 Model selection detection	18](#_Toc221806650)

[S5.3 Model selection survival	19](#_Toc221806651)

[References	20](#_Toc221806652)

# S1: Vegetation survey

## S1.1 Methods

Vegetation surveys were conducted in autumn 2022 at the three squirrel study sites in Berlin (urban park, urban cemetery, woodland area) to characterise structural and compositional differences among sites. At each site, six circular plots (radius 5 m) were randomly assigned using a grid-based approach in QGIS. Plots without tree presence were excluded and replaced to ensure relevance for an arboreal species.

Within each plot, all woody plants were recorded and identified to species or genus level where possible. For each plot, the following variables were quantified: woody plant diversity (Hill numbers q0–q2), diameter at breast height (DBH) of trees, basal area, number of broad-leaved and coniferous trees, canopy closure, deadwood share, rejuvenation percentage, and tree vitality. DBH was measured at 1.3 m height and basal area calculated per plot. Canopy closure, deadwood share, rejuvenation, and vitality were estimated using categorical field protocols commonly applied in forest and urban vegetation surveys.

Plants were classified as feeding plants for squirrels based on an extensive literature review of documented food resources (Table S1). Each recorded woody plant taxon was assigned to a binary category (feeding plant: yes/no), and feeding plant abundance per plot was summarised into ordinal categories for analysis.

To account for anthropogenic influences on vegetation structure, distances from each vegetation plot to the nearest footpath, street, and public transport station were calculated using OpenStreetMap data and spatial analyses in R. These distance metrics were used to assess correlations between vegetation characteristics and proximity to human infrastructure.

Vegetation data were analysed using non-metric multidimensional scaling (NMDS) based on Gower dissimilarities to integrate continuous and categorical variables. Environmental fitting was applied to identify vegetation variables contributing most strongly to ordination structure. Differences in vegetation characteristics among study sites were tested using PERMANOVA. Mantel tests were used to assess correlations between vegetation dissimilarity and distance matrices representing anthropogenic infrastructure.

Table S1: Feeding plants of the Eurasian red squirrel (Sciurus vulgaris)

| Scientific species name | English common name | German common name | nutrition resource | Source |
| --- | --- | --- | --- | --- |
| *Acer sp.* | Maple sp. | Ahorn | maple seeds, flowers | (Shuttleworth, 1997; L. Wauters et al., 1992) |
| *Aesculus sp.* | Horse chestnut sp. | Kastanie | chestnuts | (Ortiz & Muchlinski, 2015; L. Wauters et al., 1992) |
| *Berberis vulgaris* | Common barberry | Gewöhnliche Berberitze | fruit | (Ortiz & Muchlinski, 2015; L. Wauters et al., 1992) |
| *Carpinus sp.* | Hornbeam sp. | Hainbuche | hornbeam seeds | (L. A. Wauters et al., 2001) |
| *Cornus sp.* | Dogwood sp. | Hartriegel | fruit | (Ortiz & Muchlinski, 2015) |
| *Corylus colurna* | Turkish hazel | Baum-Hasel | hazelnuts | (L. Wauters et al., 1992) |
| *Fagus sp.* | Beech sp. | Buchen Arten | beechnuts | (L. Wauters et al., 1992) |
| *Fragaria sp.* | Wild strawberry | (Wald-)Erdbeere | berries | (Ortiz & Muchlinski, 2015) |
| *Juglans regia* | Walnut | Walnuss | walnuts, catkin | (Moller, 1983) |
| *Larix sp.* | Larch sp. | Lärche | cones | (L. Wauters et al., 1992) |
| *Picea sp.* | Spruce sp. | Fichte | seeds | (Moller, 1983) |
| *Pinus sp.* | Pine sp. | Kiefer | seeds, cones (male and female), Buds, male flowers, shoots | (Moller, 1983; Ortiz & Muchlinski, 2015; L. Wauters et al., 1992) |
| *Platanus sp.* | Plane tree sp. | Platane | fruit | (Krauze-Gryz & Gryz, 2015) |
| *Prunus sp.* | Stone fruit plants | Steinobstgewächse | flowers | (Krauze-Gryz & Gryz, 2015) |
| *Quercus sp.* | Oak sp. | Eiche | acorns, flowers, buds, leaves, catkin | (Ortiz & Muchlinski, 2015; L. Wauters et al., 1992) |
| *Rosa sp.* | Rose sp. | Rose | rose hips | (Ortiz & Muchlinski, 2015) |
| *Rubus fruticosus agg.* | Blackberry | Brombeere | berries | (L. Wauters et al., 1992) |
| *Sequoia sp.* | Redwood sp. | Mammutbaum | female cone | (Krauze-Gryz & Gryz, 2015) |
| *Taxus bacatta* | Yew | Eibe | fruity part of berries | (Haigh et al., 2015) |
| *Ulmus sp.* | Elm sp. | Ulme | elm seeds | (Krauze-Gryz & Gryz, 2015) |
| *Vaccinium myrtillus* | Bilberry | wilde Heidelbeeren | berries | (L. Wauters et al., 1992) |

## S1.2 Results

Vegetation characteristics differed markedly among study sites along the urbanisation gradient. NMDS ordination revealed a clear separation of plots by site, with an acceptable stress value indicating a reliable representation of multivariate dissimilarities (Figure S1). PERMANOVA confirmed significant differences in vegetation characteristics among sites (p = 0.001).

**B**

**A**

Figure S1: A) Distribution of the NMDS (grey dots) with influence of the response variables (blue dots and arrows) distinguished between R² values below 0.5 (lower influence, light blue arrows) and R² values above 0.5 (higher influence, dark blue arrows). The following abbreviations were used for the response variables: “FPA” for feeding plant amount, “av_DBH” for average DBH in m, “av_BA” for average basal area in m², “q0 – q2” for the Hill numbers q0 – q2, “Nr_Con” for number of conifers and “Nr_BLT” for number of broad-leaf trees. “Vit_lvl” indicates the average vitality level per plot, “CC” the canopy closure and “D_s” stands for the deadwood share in addition to “Rejuv” depicting the rejuvenation in percentage. B) NMDS of vegetation characteristics with depicted explanatory variables "Locations" and "Distances to path"

Environmental fitting indicated that average DBH was the strongest contributor to vegetation differentiation among plots (Table S2), followed by deadwood share and basal area. The number of broad-leaved trees and feeding plant abundance also contributed substantially to ordination structure, whereas woody plant diversity indices (Hill numbers q0-q2) explained comparatively less variation.

Vegetation in the urban park with the highest urbanisation level was characterised by relatively large trees, light canopy cover, low rejuvenation, and minimal deadwood. The medium-urbanised cemetery site showed the highest average basal area and DBH, low canopy cover, no deadwood, and no natural regeneration, while exhibiting comparatively high woody plant diversity. Feeding plants were present at low frequencies within survey plots at this site.

The woodland area representing low urbanisation was characterised by smaller average DBH and basal area but showed the highest proportion of feeding plants, substantial deadwood availability, ongoing regeneration, and a loose canopy cover. Vegetation structure at this site was strongly influenced by a high proportion of broad-leaved trees, with conifers also contributing to stand composition.

Anthropogenic distance analyses revealed that only distance to footpaths was significantly correlated with vegetation characteristics, whereas distances to streets and public transport stations showed no significant associations. The strongest effects of path proximity on vegetation were observed in the woodland area, consistent with forest management and recreational trail maintenance shaping local vegetation structure.

Overall, the vegetation analysis demonstrated pronounced structural and compositional differences among study sites, providing an ecological framework for interpreting habitat variation along the urbanisation gradient.

Table S2: Results of the environmental fitting analysis of the response variables from the NMDS.

| Response variable | R^2^ |
| --- | --- |
| Average dbh | 0.747 |
| Deadwood share | 0.641 |
| Average basal area | 0.614 |
| Number of broadleaf trees | 0.587 |
| Feeding plant amount | 0.583 |
| Average vitality level | 0.523 |
| Canopy closure | 0.473 |
| Rejuvenation percentage | 0.450 |
| Number of conifers | 0.423 |
| q2 | 0.351 |
| q1 | 0.270 |
| q0 | 0.191 |

# S2: Human activity index (HAI)

Additional information

“Other activities” included humans not directly observable as pedestrians (e.g. individuals in vehicles) or those generating elevated noise due to carried objects (e.g. suitcases or leaf blowers).

We recorded ambient temperature, cloud cover (%), and general weather conditions during each observation period to document sampling conditions, but they were not included as covariates in subsequent analyses.

Figure S2: Human activity index (HAI) per study site

# S3: CMR

## S3.1 Detailed description of the CMR-process

All applicable international, national, and institutional guidelines for the care and use of animals were followed. All handling of the animals was carried out with the approval of the Berlin State Office for Health and Social Affairs (LaGeSO) and in compliance with the Animal Welfare Act (§8 Article 1) under number G0020/21. The principle underlying the data collection in this study is a statistical method known as capture-mark-recapture (CMR). CMR is used to estimate population sizes of mobile animals by marking individuals in an initial capture session and then quantifying the proportion of marked individuals in subsequent recapture sessions. In our study, squirrels were captured, marked, and in the best case, recaptured in a total of eight project phases.

We captured squirrels biannually in spring and autumn using Tomahawk Live Traps (Tomahawk Live Trap, model 103 and 104, Hazelhurst, USA). At each study site 10 traps were randomly distributed in the forested parts of the study areas (Figure 1 in the main text), preferably in places concealed by vegetation and covering areas of similar size and set up at the bottom or in trees at a maximum height of 1.5 meters. Two weeks before the start of the respective field season we brought the traps to the study sites and pre-baited them with walnuts so that the squirrels would become accustomed to their presence. New walnuts were added every 2-3 days. Traps were only relocated within the study site due to infrastructural changes or a lack of trapping success. Any moved traps were given a new name. To be able to compare trapping success across sites, the trapping effort was kept as standardized as possible with five days spent at each study site per season. During each field season there was one camera trap (Anton Seissiger GmbH, Seissiger Special-Cam 3, Würzburg, Germany) facing each live trap. These camera traps worked with a motion detector and took photos every time a living being moved within the focus area with a pause of 10 seconds after each photo.

Field work started at study sites Rahnsdorf and Treptower Park in autumn 2021 and in Lilienthal in autumn 2022. We activated a maximum of 10 traps at one study site in the morning between 6-8am and closed around 12pm during each trapping session. We checked them regularly, at least every 30 minutes. If a squirrel was in the trap (Figure S3A), we covered it with a towel or similar. Then we transferred the individuals into a handling cone (Figure S3B) to ensure safe handling. At the beginning we checked if it was a new capture or a recapture with an ID reader (LID 573 Multi-Chip Pocket Reader, Euro I.D.; Figure S3C) that recognizes previously scanned chips. If the squirrel was unknown, we chipped it with an individual ID transponder (EURO I.D., transponder ID 100, Frechen/Köln, Germany) and took ear tissue (2mm^2^), fur, oral (Figure S3D), anal and fecal samples. In addition, we measured its body length, tail length, hind foot length, and weight, and determined the sex (Figure S3E), nutritional and physical condition, and provided a docility score depending on its behavior in the trap, in the cone and during release (e.g., quiet, calm, agitated, vocalizing, fighting). After all handling procedures we released the squirrel (Figure S3F). The duration of this process was approx. 15 to 20 minutes. We stored all samples at -‑20°C. The genetic samples were stored at this temperature for a few days (max. 7 days) and then stored at -‑80°C until analysis.

Figure S3: Details of the capture and handling procedure. (A) Squirrel in a Tomahawk live trap. (B) Individual placed in a handling bag during weighing using a spring scale. (C) Identification of recaptured individuals using a PIT tag reader. (D) Collection of an oral swab. (E) Sex determination; the individual shown is male. (F) Release of the squirrel at the capture site. Pictures by Jon A. Juárez.

## S3.2 Results of capturing and sampling

Figure S4: Capturing events of squirrels per study site.

Figure S5: Relationship between weight (y-axis) and body length (x-axis) of squirrels across the study sites.

Figure S6: Number of captured individuals per study site.

Figure S7: Sex ratio of captured squirrels per study site.

Figure S8: N umber of tissue samples for the genetic analysis per study site.

# S4: Genetic analyses

## S4.1 DNA extraction, genotyping and genotyping error estimation

DNA was extracted from ear tissue samples using the “First-DNA all-tissue Kit” (Gen-Ial GmbH, Troisdorf, Germany), following the manufacturer’s instructions. We measured DNA concentra-tions using a NanoDrop1000 (PeqLab GmbH, Erlangen Germany). We used 16 microsatellite loci for the genotyping (Todd 2000, Hale et al. 2001). Loci were amplified in four multiplex PCRs using the Qiagen Multiplex PCR Kit (Qiagen, Germany). Each reaction contained 1× QIAGEN Multiplex Master Mix and 0.1-0.3 μmol of each primer. Additionally, 1× Q-solution (Qiagen) was added to Multiplex 4. An amount of 100 ng DNA was used for each PCR. PCRs were started with a 5-min denaturation at 95°C, followed by 35 cycles of denaturing at 94°C for 30 s, annealing at 63°C- 55°C (cycle 1: 63°, cycle 2: 61°, cycle 3: 59°, cycle 4: 57° and for the remaining 31 cycles: 55°) for 30 s and extension at 72°C for 30 s. The reaction was terminated after a final extension at 60°C for 30 min. Cycling was performed on a peqSTAR96 Universal Gradient thermocycler (Peqlab, Erlangen, Germany), and fragments (al-leles) were separated on an A3130xl automated DNA sequencer (ABI Applied Biosystems, Fos-ter, CA, USA). Allele sizes were called based on a GeneScanTM 500 ROXTM dye size calibra-tion standard using GENEMAPPER v.3.7 (ABI).

Afterwards we applied a genotyping error estimation process: We tested for null alleles, large allele dropouts and the Hardy-Weinberg-Equilibrium (HWE) with Micro-Checker v.2.2.3 (Van Oosterhout et al., 2004). We applied a threshold of 0.20 for the Van Oosterhout p-value. We used CERVUS v.3.0.7 (Kalinowski et al., 2007, 2010; Marshall et al., 1998) to calculate allele frequencies across all 16 loci and to conduct an identity analysis for detecting matching genotypes within our dataset. We assumed that two samples originate from the same individual if they have been identified with the same chip ID or they had matching genotypes. We allowed two mismatching alleles in two loci with an allelic size difference of 4 or less. Additionally, the samples had to be from individuals that were of the same sex and from the same study site. In cases where two samples matched, we removed the newest one if all 16 loci matched. If 15 or 14 loci matched, we retained the heterozygous sample when possible, as heterozygous genotypes provide more allelic information and are thus more informative for subsequent analyses. If two samples from the same individual matched with small differences in the alleles and the samples are e.g. homozygous with different values, we generated a new genotype that is heterozygous and consists of the two homozygous ones. These genotypes are marked in all tables.

After removing one sample from each matching pair, we re-tested with Micro-Checker. We checked all pairs of loci for linkage disequilibrium including Bonferroni correction for multiple comparisons with Arlequin v.3.5.2.2 (Excoffier & Lischer, 2010). For 16 loci, 120 comparisons were possible, resulting in a p-value with Bonferroni correction of 0.0004167. After testing for linkage disequilibrium, 2 loci (Rsu3 and Scv12) showed significant deviations or were flagged due to technical issues (e.g. frequent amplification failure) for all individuals. Therefore STRUCTURE analyses were conducted both with and without these loci. However, comparisons conducted separately for each study site did not reveal a consistent pattern of linkage disequilibrium across loci, indicating that the observed signals were not systematic and unlikely to reflect true linkage disequilibrium.

In addition, 3 individuals (2 from the urban park, 1 from the woodland area) had missing data at 3 loci. We therefore ran the STRUCTURE analyses with 133 individuals (without the 2 from urban park) and also with 132 individuals (additionally without the squirrel from woodland area). Consequently, we tested 6 data sets with STRUCTURE: 136, 134, 133 individuals and with 16 and 14 loci respectively. We tested it with 14 loci, even though this pattern was not uniform, to see if differences could still be detected. As no significant differences were detected in the results, we present the results based on all microsatellite loci and used them in all further analyses. In addition, the linkage imbalance was tested for each study location to determine whether there were any discernible patterns at each study location, but this was not the case. We calculated pairwise F_ST_ values to assess the degree of differentiation between study site groups, not between the clusters. F_ST_ measures genetic differentiation among subpopulations relative to the total population.

## S4.2 Information on microsatellite markers

Table S3: Properties of the microsatellite loci used in this study. Multiplex: indicates in which multiplex reaction the primer pair was used. N = number of samples successfully analysed, in total we have 158 samples. F= forward primer; R = reverse primer.

| Locus | Multiplex | Primer sequence (5’-3’) | Dye | N | Length (bp) | Literature |
| --- | --- | --- | --- | --- | --- | --- |
| Rsu3 | 1 | F: GCCAAAATCTAGCCCAAGAAG  R: CTCAGGTGTGGGAAAGAAGC | 6-Fam | 156 | 161-173 | (Todd, 2000) |
| Rsu5 | 1 | F: CCCAGTCTACATTAAAGGGC  R: GCCTATACACTATAATTGACTG | Hex | 156 | 123-143 | (Todd, 2000) |
| Scv1 | 1 | F: CTCCTCTTCCAAGGGTGACA  R: GATGGCCTCTGTTTCTCTGC | Atto550 | 157 | 134-183 | (Hale et al., 2001) |
| Rsu4 | 2 | F: CAATCCTCCCATCCTGCTGC  R: TAGGCAGTCAGATAGGTGGG | 6-Fam | 158 | 256-284 | (Todd, 2000) |
| Rsu6 | 2 | F: GGCATAGGGCACGTGAAG  R: GGGCCAATCTCATACCAAG | 6-Fam | 158 | 122-131 | (Todd, 2000) |
| Scv12 | 2 | F: ACAGAGACTTCCACGTGTGC  R: GCCTCCCCAAGAGAAAAATC | 6-Fam | 155 | 190-208 | (Hale et al., 2001) |
| Scv23 | 2 | F: AAACACCTGAGACAGGCAAC  R: GTGTTTGGCAATGTCAGCTC | Hex | 155 | 151-167 | (Hale et al., 2001) |
| Scv13 | 3 | F: CTGGGTTCAATCCCAATGTC  R: ATAGATGCACCCTGAAGCTG | Hex | 158 | 167-181 | (Hale et al., 2001) |
| Scv32 | 3 | F: CACCATGAGCCTTCCAGACT  R: ACCTCTGCCCACCCATTT | Hex | 157 | 234-244 | (Hale et al., 2001) |
| Scv8 | 3 | F: TCTTATGGCCAGCCTGTCTT  R: GGAATGGAGGTGGTTGGTAA | 6-Fam | 157 | 192-200 | (Hale et al., 2001) |
| Scv9 | 3 | F: TCATGCTAATGCCAATCACAA  R: TGGAAGTTCTGTGGACGTTTC | Atto550 | 158 | 190-218 | (Hale et al., 2001) |
| Rsu1 | 4 | F: CTGGGTTCACTGACTTCTCC  R: CACTCTCAGAGGCCAAAGTC | Hex | 158 | 172-196 | (Todd, 2000) |
| Scv14 | 4 | F: AGGTGCACTGATATGCATCG  R: GGTCAGCTTTGTGGCTTTGT | 6-Fam | 152 | 192-206 | (Hale et al., 2001) |
| Scv16 | 5 | F: GGGAAGAACTGGGGAATAACA  R: TTTCCCTTTCCCATTCTCCT | 6-Fam | 158 | 181-219 | (Hale et al., 2001) |
| Scv31 | 5 | F: CCAAGTTCCAGACCAACCTC  R: TCGGGTCTCTAAGGAGATGG | Atto550 | 154 | 179-201 | (Hale et al., 2001) |
| Scv6 | 5 | F: GCAATCCTTGTCCTTGCATT  R: TGAGTCATTGGATGAAAACCA | Hex | 158 | 185-201 | (Hale et al., 2001) |

## S4.3 Results of genetic analysis per locus

Table S4: Locus-level genetic diversity measures. Null = oosterhout statistic for null alleles, N_A_ = Number of alleles, A_R_ = Allelic richness, PA = private alleles, H_O_ = observed heterozygosity, H_E_ = Expected heterozygosity, A_E_ = Effective number of alleles, F_IS_ = Inbreeding coefficient. U = urban park, C = cemetery, W = Woodland area, T = Total

| Locus | Null | N_A_ | | | | A_R_ | | | | PA | | | | H_O_ | | | | H_E_ | | | | A_E_ | | | F_IS_ | | |
| --- | --- | --- | --- | --- | --- | --- | --- | --- | --- | --- | --- | --- | --- | --- | --- | --- | --- | --- | --- | --- | --- | --- | --- | --- | --- | --- | --- |
| Study site | Total | U | C | W | T | U | C | W | T | U | C | W | T | U | C | W | T | U | C | W | T | U | C | W | U | C | W |
| Rsu3 | -0.043 | 3 | 3 | 3 | 3 | 3.00 | 3.00 | 3.00 | 3.00 | 0 | 0 | 0 | 0 | 0.65 | 0.56 | 0.80 | 0.64 | 0.57 | 0.61 | 0.61 | 0.60 | 2.32 | 2.55 | 2.53 | -0.14 | 0.08 | -0.32 |
| Rsu5 | 0.150 | 3 | 4 | 4 | 5 | 2.53 | 3.62 | 3.95 | 4.97 | 0 | 1 | 1 | 2 | 0.10 | 0.32 | 0.35 | 0.22 | 0.12 | 0.45 | 0.58 | 0.35 | 1.14 | 1.82 | 2.39 | 0.22 | 0.29 | 0.40 |
| Scv1 | 0.024 | 5 | 4 | 5 | 5 | 4.17 | 4.00 | 4.95 | 5.00 | 0 | 0 | 0 | 0 | 0.39 | 0.90 | 0.40 | 0.58 | 0.44 | 0.74 | 0.51 | 0.61 | 1.79 | 3.90 | 2.06 | 0.11 | -0.21 | 0.22 |
| Rsu4 | 0.071 | 6 | 6 | 7 | 9 | 5.30 | 5.34 | 7.00 | 9.00 | 2 | 0 | 1 | 3 | 0.65 | 0.64 | 0.70 | 0.65 | 0.70 | 0.73 | 0.80 | 0.76 | 3.38 | 3.70 | 5.00 | 0.08 | 0.12 | 0.13 |
| Rsu6 | 0.004 | 4 | 2 | 4 | 5 | 3.29 | 2.00 | 4.00 | 4.96 | 1 | 0 | 1 | 2 | 0.63 | 0.38 | 0.45 | 0.51 | 0.60 | 0.36 | 0.52 | 0.52 | 2.51 | 1.56 | 2.07 | -0.05 | -0.06 | 0.13 |
| Scv12 | 0.057 | 3 | 3 | 3 | 3 | 3.00 | 2.97 | 3.00 | 3.00 | 0 | 0 | 0 | 0 | 0.57 | 0.51 | 0.37 | 0.52 | 0.64 | 0.54 | 0.36 | 0.59 | 2.81 | 2.18 | 1.57 | 0.12 | 0.06 | -0.02 |
| Scv23 | 0.003 | 3 | 3 | 3 | 3 | 3.00 | 3.00 | 3.00 | 3.00 | 0 | 0 | 0 | 0 | 0.53 | 0.53 | 0.85 | 0.58 | 0.59 | 0.52 | 0.66 | 0.58 | 2.47 | 2.08 | 2.97 | 0.11 | -0.02 | -0.28 |
| Scv13 | 0.048 | 3 | 4 | 5 | 5 | 2.49 | 3.29 | 4.95 | 5.00 | 0 | 0 | 0 | 0 | 0.20 | 0.28 | 0.50 | 0.27 | 0.21 | 0.33 | 0.49 | 0.30 | 1.26 | 1.49 | 1.98 | 0.04 | 0.15 | -0.01 |
| Scv32 | 0.067 | 12 | 11 | 10 | 18 | 9.08 | 9.12 | 10.00 | 17.92 | 3 | 1 | 3 | 7 | 0.78 | 0.72 | 0.79 | 0.76 | 0.87 | 0.80 | 0.80 | 0.88 | 7.94 | 4.94 | 4.89 | 0.10 | 0.10 | 0.01 |
| Scv8 | 0.041 | 4 | 4 | 5 | 6 | 3.89 | 3.95 | 5.00 | 6.00 | 1 | 0 | 0 | 1 | 0.38 | 0.52 | 0.53 | 0.46 | 0.39 | 0.57 | 0.54 | 0.50 | 1.65 | 2.34 | 2.15 | 0.02 | 0.09 | 0.02 |
| Scv9 | 0.033 | 4 | 5 | 3 | 5 | 3.88 | 4.24 | 3.00 | 4.96 | 0 | 1 | 0 | 1 | 0.72 | 0.56 | 0.55 | 0.64 | 0.68 | 0.66 | 0.59 | 0.69 | 3.09 | 2.91 | 2.47 | -0.07 | 0.15 | 0.08 |
| Rsu1 | 0.079 | 6 | 4 | 4 | 6 | 5.96 | 3.77 | 3.95 | 6.00 | 2 | 0 | 0 | 2 | 0.74 | 0.50 | 0.70 | 0.64 | 0.81 | 0.65 | 0.62 | 0.76 | 5.26 | 2.82 | 2.63 | 0.09 | 0.23 | -0.13 |
| Scv14 | 0.171 | 4 | 5 | 5 | 7 | 3.28 | 4.37 | 4.95 | 7.00 | 1 | 1 | 1 | 3 | 0.13 | 0.41 | 0.55 | 0.30 | 0.31 | 0.57 | 0.72 | 0.49 | 1.45 | 2.32 | 3.55 | 0.57 | 0.28 | 0.23 |
| Scv16 | 0.010 | 3 | 4 | 5 | 7 | 2.94 | 3.83 | 4.90 | 6.91 | 0 | 2 | 2 | 4 | 0.58 | 0.60 | 0.50 | 0.58 | 0.55 | 0.60 | 0.64 | 0.59 | 2.23 | 2.50 | 2.81 | -0.06 | -0.00 | 0.22 |
| Scv31 | 0.197 | 11 | 8 | 10 | 15 | 9.25 | 7.39 | 9.94 | 14.98 | 2 | 0 | 4 | 6 | 0.56 | 0.38 | 0.75 | 0.53 | 0.87 | 0.81 | 0.87 | 0.88 | 7.52 | 5.32 | 7.67 | 0.35 | 0.53 | 0.14 |
| Scv6 | 0.007 | 6 | 6 | 8 | 8 | 4.77 | 5.32 | 8.00 | 8.00 | 0 | 0 | 2 | 0 | 0.63 | 0.74 | 0.85 | 0.70 | 0.63 | 0.71 | 0.85 | 0.71 | 2.67 | 3.50 | 6.55 | -0.01 | -0.04 | -0.00 |
| Mean | / | 5.00 | 4.75 | 5.25 | 6.88 | 4.36 | 4.33 | 5.22 | 6.86 | / | / | / | / | 0.52 | 0.53 | 0.60 | 0.54 | 0.56 | 0.60 | 0.64 | 0.61 | 3.09 | 2.87 | 3.33 | 0.09 | 0.11 | 0.05 |
| Total | / | 80 | 76 | 84 | 110 | / | / | / | / | 12 | 6 | 15 | 33 | / | / | / | / | / | / | / | / | / | / | / | / | / | / |

We provide detailed locus-specific statistics for each study site in Table S4. Across all 16 microsatellite loci, each population harbored multiple alleles, with some alleles being common (frequency > 0.5) and others rare or absent (frequency = 0). N_A_ per study site was not significantly different (Kruskal-Wallis test: χ² = 0.55, df = 2, p = 0.76). Same for A_R_ (χ² = 3.23, df = 2, p = 0.20). Each study site also contained private alleles, i.e., alleles found exclusively in individuals from that site: 11 in the urban park, 6 in the cemetery, and 6 in the woodland area, indicating some degree of population differentiation.

Observed and expected heterozygosity did not differ significantly among the three study sites. Mean observed heterozygosity (H_O_) was 0.60 in Rahnsdorf, 0.52 in Treptower Park, and 0.53 in Lilienthal (χ² = 1.06, df = 2, p = 0.59). Mean expected heterozygosity (H_E_) was 0.64, 0.56, and 0.53, respectively (χ² = 0.54, df = 2, p = 0.76), indicating comparable levels of genetic diversity across populations.

On the other side there are differences between H_O_ and H_E_ within populations. In the woodland area, H_O_ did not differ significantly from H_E_ (paired t-test, t = -1.12, df = 15, p = 0.28), indicating no deviation from Hardy-Weinberg expectations. In the urban park, H_O_ tended to be slightly lower than H_E_, but the difference was not statistically significant (t = -1.97, df = 15, p = 0.067). In Lilienthal, however, H_O_ was significantly lower than H_E_ (t = -2.20, df = 15, p = 0.044).

Standardized multilocus heterozygosity (sMLH) differed among populations (Kruskal-Wallis test: χ² = 6.55, df = 2, p = 0.038). Post-hoc Dunn tests with Bonferroni correction showed that squirrels from Treptower Park had significantly lower sMLH than those from Rahnsdorf (adj. p = 0.028), while comparisons involving Lilienthal were not significant (all adj. p > 0.05). These results suggest subtle differences in individual-level genetic diversity across populations.

The effective number of alleles (A_E_) also differed slightly among populations. Rahnsdorf exhibited the highest mean A_E_ per locus (3.33 ± 1.77), Treptower Park showed similar mean A_E_ (3.09 ± 2.06) but with higher locus-to-locus variation, and Lilienthal had the lowest mean A_E_ (2.87 ± 1.13), reflecting lower overall diversity and more uniform allele distribution, which could indicate reduced gene flow or mild isolation.

Inbreeding coefficients (F_IS_) were low to moderate across populations. Mean F_IS_ values were 0.05 (95% CI: −0.03–0.13) for the woodland area, 0.09 (95% CI: 0.02–0.18) for the urban park, and 0.11 (95% CI: 0.03–0.19) for the cemetery. Confidence intervals for the urban park and cemetery did not include zero, indicating significant heterozygote deficits. Overall, F_IS_ increased slightly along the urbanization gradient, consistent with potential population substructure, reduced gene flow, or mild inbreeding in more urbanized or isolated areas. The individual inbreeding coefficient (F) did not sho any significant differences (Kruskal-Wallis test: χ² = 3.35, df = 2, p = 0.19).

## S4.4: Results of population genetic structure analysis

Table S5: Result table of the Cluster analysis with all individuals (N = 135). MEAN LN PROB = mean estimated ln probability of data. SD mean LN prob = standard deviation. LNK1 = First derivate. LNK2 = Second derivate. ΔK = Third derivate.

| K | mean ln prob | SD mean LN prob | lnK1 | lnK2 | ΔK |
| --- | --- | --- | --- | --- | --- |
| 1 | -5483.00 | 0.43 | NA | NA | NA |
| 2 | -5208.87 | 5.97 | 274.14 | 142.84 | 23.94 |
| 3 | -5077.58 | 9.41 | 131.29 | 27.42 | 2.91 |
| 4 | -4973.70 | 11.56 | 103.88 | 28.56 | 2.47 |
| 5 | -4898.39 | 51.92 | 75.31 | 44.66 | 0.86 |
| 6 | -4867.74 | 129.09 | 30.65 | 10.65 | 0.08 |
| 7 | -4847.74 | 91.95 | 20.00 | 73.50 | 0.80 |
| 8 | -4901.24 | 173.39 | -53.50 | 91.32 | 0.53 |
| 9 | -4863.42 | 151.04 | 37.82 | 232.65 | 1.54 |
| 10 | -5058.25 | 487.26 | -194.83 | NA | NA |

Figure S9: Determination of the most likely number of genetic clusters (K) using the Evanno method (Evanno et al., 2005) based on STRUCTURE analyses for all individuals (N = 135). A) Mean estimated ln probability of data ± SD. B) First derivate ± SD. C) Second derivate ± SD. D) ΔK. On the x-axis you can always see the number of clusters (K).

Table S6: Result table of the Cluster analysis without closely related individuals (N = 100). MEAN LN PROB = mean estimated ln probability of data. SD mean LN prob = standard deviation. LNK1 = First derivate. LNK2 = Second derivate. ΔK = Third derivate.

| k | mean ln prob | SD mean LN prob | lnk1 | lnk2 | ΔK |
| --- | --- | --- | --- | --- | --- |
| 1 | -4189.90 | 0.47 | NA | NA | NA |
| 2 | -4024.64 | 3.37 | 165.26 | 63.20 | 18.75 |
| 3 | -3922.59 | 14.65 | 102.06 | 62.64 | 4.28 |
| 4 | -3883.17 | 51.63 | 39.42 | 71.52 | 1.39 |
| 5 | -3915.28 | 116.76 | -32.11 | 67.41 | 0.58 |
| 6 | -3879.98 | 31.72 | 35.30 | 69.52 | 2.19 |
| 7 | -3914.20 | 45.88 | -34.22 | 21.41 | 0.47 |
| 8 | -3969.82 | 31.32 | -55.62 | 4.35 | 0.14 |
| 9 | -4029.79 | 106.05 | -59.97 | 44.76 | 0.42 |
| 10 | -4045.00 | 53.74 | -15.21 | NA | NA |

Figure S10: Determination of the most likely number of genetic clusters (K) using the Evanno method (Evanno et al., 2005) based on STRUCTURE analyses excluding closely related individuals (N = 100). A) Mean estimated ln probability of data ± SD. B) First derivate ± SD. C) Second derivate ± SD. D) ΔK. On the x-axis you can always see the number of clusters (K).

Pairwise F_ST_ values indicated only moderate to low differentiation between squirrels from woodland area and cemetery (F_ST_ = 0.08, 95% CI = 0.06-0.10; Table S7) and between squirrels from woodland area and urban park (F_ST_ = 0.07, 95% CI = 0.05-0.09). The lowest differentiation occurred between squirrels from cemetery and urban park (F_ST_ = 0.06, 95% CI = 0.04-0.08), suggesting ongoing gene flow between all study sites

Table S7: Above the diagonal: Pairwise F_ST_ values with Confidence Interval (CI). Below the diagonal: distance in km between the study sites

|  | Woodland area | Cemetery | Urban park |
| --- | --- | --- | --- |
| Woodland area | NA | 0.08 (0.06, 0.10) | 0.07 (0.05, 0.09) |
| Cemetery | 20 | NA | 0.06 (0.04, 0.08) |
| Urban park | 15 | 4 | NA |

# S5: Survival analysis

## S5.1 Detailed methods

To estimate survival and detection probabilities, we used Cormack-Jolly-Seber models, a capture-recapture method developed for open populations (Cooch & White, 2025). We define a capturing event as the capture of an individual during one of the designated project phases of this study. These models allow the estimation of survival probabilities between capturing events and detection probabilities based on individual capture histories. We define a capturing event as the capture of an individual during one of the designated project phases of this study. Consequently, the capture and recaptures of the same individual within a single project phase are not processed individually. Based on this the capture history of each individual is built. It is represented as a numeric string, with “1” indicating that an individual was captured during a particular project phase and “0” indicating that it was not captured (White & Burnham, 1999). In the CJS models, the survival probability Φ represents the probability that an individual survives from the time of capture 𝑖 to 𝑖 + 1, while the detection probability 𝑝 represents the probability that an individual is still alive at the time of capture 𝑖 and will be encountered (Cooch & White, 2025). In the context of our study, Φ means that a squirrel survives from one project phase to the next, while 𝑝 indicates that the squirrel is detected during a specific project phase.

## S5.2 Model selection detection

Table S8: Model list for the identification of the best predictors for detection probability

| Φ | 𝑝 | model | npar | AICc | DeltaAICc | weight | Deviance |
| --- | --- | --- | --- | --- | --- | --- | --- |
| ~1 | ~Season + Sex | Φ(~1) 𝑝 (~Season + Sex) | 4 | 463.35 | 0.00 | 0.31 | 228.17 |
| ~1 | ~Season * (Sex + trap_hours) | Φ(~1) 𝑝 (~Season * (Sex + trap_hours)) | 7 | 464.2 | 0.85 | 0.2 | 222.7 |
| ~1 | ~Sex * Season | Φ(~1) 𝑝 (~Sex * Season) | 5 | 464.37 | 1.02 | 0.18 | 227.1 |
| ~1 | ~Sex | Φ(~1) 𝑝 (~Sex) | 3 | 465.07 | 1.72 | 0.13 | 231.96 |
| ~1 | ~trap_hours + Sex | Φ(~1) 𝑝 (~trap_hours + Sex) | 4 | 466.46 | 3.11 | 0.06 | 231.28 |
| ~1 | ~Sex * Study_site | Φ(~1) 𝑝 (~Sex * Study_site) | 7 | 466.74 | 3.39 | 0.06 | 225.24 |
| ~1 | ~Season | Φ(~1) 𝑝 (~Season) | 3 | 468.82 | 5.47 | 0.02 | 235.71 |
| ~1 | ~trap_hours * Season | Φ(~1) 𝑝 (~trap_hours * Season) | 5 | 469.8 | 6.45 | 0.01 | 232.53 |
| ~1 | ~trap_hours + Season | Φ(~1) 𝑝 (~trap_hours + Season) | 4 | 470.44 | 7.09 | 0.01 | 235.26 |
| ~1 | ~1 | Φ(~1) 𝑝 (~1) | 2 | 471.21 | 7.86 | 0.01 | 240.15 |
| ~1 | ~trap_hours + Study_site | Φ(~1) 𝑝 (~trap_hours + Study_site) | 5 | 471.66 | 8.31 | 0 | 234.4 |
| ~1 | ~Study_site | Φ(~1) 𝑝 (~Study_site) | 4 | 471.99 | 8.64 | 0 | 236.81 |
| ~1 | ~trap_hours | Φ(~1) 𝑝 (~trap_hours) | 3 | 472.55 | 9.2 | 0 | 239.44 |
| ~1 | ~trap_hours * Study_site | Φ(~1) 𝑝 (~trap_hours * Study_site) | 7 | 475.6 | 12.25 | 0 | 234.1 |

## S5.3 Model selection survival

Table S9: Model list for the identification of the best predictors for survival

| Φ | 𝑝 | model | npar | AICc | DeltaAICc | weight | Deviance |
| --- | --- | --- | --- | --- | --- | --- | --- |
| ~F | ~Season + Sex | Φ(~F) 𝑝(~Season + Sex) | 5 | 463.11 | 0 | 0.2 | 452.85 |
| ~1 | ~Season + Sex | Φ(~1) 𝑝(~Season + Sex) | 4 | 463.35 | 0.24 | 0.18 | 228.17 |
| ~Sex | ~Season + Sex | Φ(~Sex) 𝑝(~Season + Sex) | 5 | 464.53 | 1.42 | 0.1 | 227.27 |
| ~MLH_sq | ~Season + Sex | Φ(~MLH_sq) 𝑝(~Season + Sex) | 5 | 464.7 | 1.58 | 0.09 | 454.43 |
| ~mean_30 | ~Season + Sex | Φ(~mean_30) 𝑝(~Season + Sex) | 5 | 464.71 | 1.6 | 0.09 | 454.45 |
| ~Season | ~Season + Sex | Φ(~Season) 𝑝(~Season + Sex) | 5 | 465.4 | 2.28 | 0.06 | 228.13 |
| ~MLH_sq + Sex | ~Season + Sex | Φ(~MLH_sq + Sex) 𝑝(~Season + Sex) | 6 | 465.61 | 2.5 | 0.06 | 453.24 |
| ~Study_site | ~Season + Sex | Φ(~Study_site) 𝑝(~Season + Sex) | 6 | 465.84 | 2.72 | 0.05 | 226.46 |
| ~MLH_sq * Sex | ~Season + Sex | Φ(~MLH_sq * Sex) 𝑝(~Season + Sex) | 7 | 466.2 | 3.08 | 0.04 | 451.7 |
| ~Sex + Season | ~Season + Sex | Φ(~Sex + Season) 𝑝(~Season + Sex) | 6 | 466.64 | 3.52 | 0.03 | 227.26 |
| ~MLH_sq + Study_site | ~Season + Sex | Φ(~MLH_sq + Study_site) 𝑝(~Season + Sex) | 7 | 467.19 | 4.07 | 0.03 | 452.69 |
| ~MLH_sq * Study_site | ~Season + Sex | Φ(~MLH_sq * Study_site) 𝑝(~Season + Sex) | 9 | 467.55 | 4.44 | 0.02 | 448.75 |
| ~Sex * Season | ~Season + Sex | Φ(~Sex * Season) 𝑝(~Season + Sex) | 7 | 468.11 | 5 | 0.02 | 226.61 |
| ~Sex + Season + Study_site | ~Season + Sex | Φ(~Sex + Season + Study_site) 𝑝(~Season + Sex) | 8 | 469.33 | 6.22 | 0.01 | 225.69 |
| ~Study_site * (Sex + Season) | ~Season + Sex | Φ(~Study_site * (Sex + Season)) 𝑝(~Season + Sex) | 12 | 469.39 | 6.28 | 0.01 | 216.97 |
| ~Sex + F + Study_site + mean_30 + Season | ~Season + Sex | Φ(~Sex + F + Study_site + mean_30 + Season) 𝑝(~Season + Sex) | 10 | 471.31 | 8.19 | 0 | 450.31 |
| ~mean_30 * Study_site | ~Season + Sex | Φ(~mean_30 * Study_site) 𝑝(~Season + Sex) | 9 | 472.27 | 9.16 | 0 | 453.46 |
| ~Sex + MLH_sq + Study_site + mean_30 + Season | ~Season + Sex | Φ(~Sex + MLH_sq + Study_site + mean_30 + Season) 𝑝(~Season + Sex) | 10 | 472.6 | 9.49 | 0 | 451.61 |
| ~Season * (Sex + F + Study_site + mean_30) | ~Season + Sex | Φ(~Season * (Sex + F + Study_site + mean_30)) 𝑝(~Season + Sex) | 15 | 478.33 | 15.22 | 0 | 446.12 |
| ~Season * (Sex + MLH_sq + Study_site + mean_30) | ~Season + Sex | Φ(~Season * (Sex + MLH_sq + Study_site + mean_30)) 𝑝(~Season + Sex) | 15 | 479.3 | 16.18 | 0 | 447.08 |

Figure S 11: Apparent survival probability in relation to the individual inbreeding coefficient F.

Table S10: Model estimates on the logit scale of the best squirrel survival model. Study sites were denoted as groups. SE = Standard error. CI = Confidence interval. The intercept represents the following levels of the factor variables: Season = Autumn; Sex = females

| Parameter | Estimate | SE | Lower CI | Upper CI |
| --- | --- | --- | --- | --- |
| Survival model | | | | |
| Φ _intercept_ | 0.47 | 0.35 | -0.21 | 1.16 |
| Φ _f_ | 2.19 | 1.51 | -0.77 | 5.15 |
|  |  |  |  |  |
| Detection model | | | | |
| P intercept | 0.51 | 0.33 | -0.14 | 1.16 |
| P season (spring) | -0.65 | 0.34 | -1.32 | 0.01 |
| P sex (males) | 1.02 | 0.39 | 0.26 | 1.79 |

# References

Cooch, E. G., & White, G. C. (2025). *Program MARK - A Gentle Introduction* (26th edn). http://www.phidot.org/software/mark/docs/book/

Excoffier, L., & Lischer, H. E. L. (2010). Arlequin suite ver 3.5: A new series of programs to perform population genetics analyses under Linux and Windows. *Molecular Ecology Resources*, *10*(3), 564–567. https://doi.org/10.1111/j.1755-0998.2010.02847.x

Haigh, A., O’Riordan, R., & Butler, F. (2015). The preference for yew (Taxus baccata) by a red (Sciurus vulgaris) only squirrel population. *Wildlife Research*, *42*(5), 426. https://doi.org/10.1071/WR15009

Hale, M. L., Bevan, R., & Wolff, K. (2001). New polymorphic microsatellite markers for the red squirrel ( *Sciurus vulgaris* ) and their applicability to the grey squirrel ( *S. carolinensis* ). *Molecular Ecology Notes*, *1*(1–2), 47–49. https://doi.org/10.1046/j.1471-8278.2000.00020.x

Kalinowski, S. T., Taper, M. L., & Marshall, T. C. (2007). Revising how the computer program cervus accommodates genotyping error increases success in paternity assignment. *Molecular Ecology*, *16*(5), 1099–1106. https://doi.org/10.1111/j.1365-294X.2007.03089.x

Kalinowski, S. T., Taper, M. L., & Marshall, T. C. (2010). Corrigendum. *Molecular Ecology*, *19*(7), 1512–1512. https://doi.org/10.1111/j.1365-294X.2010.04544.x

Krauze-Gryz, D., & Gryz, J. (2015). A review of the diet of the red squirrel (Sciurus vulgaris) in different types of habitats. In C. Shuttleworth, P. W. W. Lurz, & M. Hayward (Eds), *Red squirrels :ecology, conservation & management in Europe*. European Squirrel Initiative. https://www.researchgate.net/publication/283308835_2_A_review_of_the_diet_of_the_red_squirrel_Sciurus_vulgaris_in_different_types_of_habitats

Marshall, T. C., Slate, J., Kruuk, L. E. B., & Pemberton, J. M. (1998). Statistical confidence for likelihood‐based paternity inference in natural populations. *Molecular Ecology*, *7*(5), 639–655. https://doi.org/10.1046/j.1365-294x.1998.00374.x

Moller, H. (1983). Foods and foraging behaviour of Red (Sciurus vulgaris) and Grey (Sciurus carolinensis) squirrels. *Mammal Review*, *13*(2–4), 81–98. https://doi.org/10.1111/j.1365-2907.1983.tb00270.x

Ortiz, J. L., & Muchlinski, A. E. (2015). Food Selection of Coexisting Western Gray Squirrels and Eastern Fox Squirrels in a Native California Botanic Garden in Claremont, California. *Bulletin, Southern California Academy of Sciences*, *114*(2), 98–103. https://doi.org/10.3160/0038-3872-114.2.98

Shuttleworth, C. (1997). The effect of supplemental feeding on the diet, population density and reproduction of red squirrels. In J. Gurnell & P. W. W. Lurz (Eds), *The Conservation of Red Squirrels (Sciurus vulgaris L.)* (pp. 13–24). Peoples Trust for Endangered Species.

Todd, R. (2000). Microsatellite loci in the Eurasian red squirrel, *Sciurus vulgaris* L. *Molecular Ecology*, *9*(12), 2165–2166. https://doi.org/10.1046/j.1365-294X.2000.10536.x

Van Oosterhout, C., Hutchinson, W. F., Wills, D. P. M., & Shipley, P. (2004). micro ‐ checker: Software for identifying and correcting genotyping errors in microsatellite data. *Molecular Ecology Notes*, *4*(3), 535–538. https://doi.org/10.1111/j.1471-8286.2004.00684.x

Wauters, L. A., Gurnell, J., Martinoli, A., & Tosi, G. (2001). Does interspecific competition with introduced grey squirrels affect foraging and food choice of Eurasian red squirrels? *Animal Behaviour*, *61*(6), 1079–1091. https://doi.org/10.1006/anbe.2001.1703

Wauters, L., Swinnen, C., & Dhondt, A. A. (1992). Activity budget and foraging behaviour of red squirrels ( *Sciurus vulgaris* ) in coniferous and deciduous habitats. *Journal of Zoology*, *227*(1), 71–86. https://doi.org/10.1111/j.1469-7998.1992.tb04345.x

White, G. C., & Burnham, K. P. (1999). Program MARK: Survival estimation from populations of marked animals. *Bird Study*, *46*(sup1), S120–S139. https://doi.org/10.1080/00063659909477239
